# Intelligent depression detection with asynchronous federated optimization

**DOI:** 10.1007/s40747-022-00729-2

**Published:** 2022-06-23

**Authors:** Jinli Li, Ming Jiang, Yunbai Qin, Ran Zhang, Sai Ho Ling

**Affiliations:** 1 College of Electronic Engineering, Guangxi Normal University, Guilin, China; 2 Business and Law School, Deakin University, Geelong, Australia; 3 Faculty of Engineering and IT, University of Technology, Sydney, Australia

**Keywords:** Depression detection, Asynchronous federated learning, CNN

## Abstract

The growth of population and the various intensive life pressures everyday deepen the competitions among people. Tens of millions of people each year suffer from depression and only a fraction receives adequate treatment. The development of social networks such as Facebook, Twitter, Weibo, and QQ provides more convenient communication and provides a new emotional vent window. People communicate with their friends, sharing their opinions, and shooting videos to reflect their feelings. It provides an opportunity to detect depression in social networks. Although depression detection using social networks has reflected the established connectivity across users, fewer researchers consider the data security and privacy-preserving schemes. Therefore, we advocate the federated learning technique as an efficient and scalable method, where it enables the handling of a massive number of edge devices in parallel. In this study, we conduct the depression analysis on the basis of an online microblog called Weibo. A novel algorithm termed as CNN Asynchronous Federated optimization (CAFed) is proposed based on federated learning to improve the communication cost and convergence rate. It is shown that our proposed method can effectively protect users' privacy under the premise of ensuring the accuracy of prediction. The proposed method converges faster than the Federated Averaging (FedAvg) for non-convex problems. Federated learning techniques can identify quality solutions of mental health problems among Weibo users.

## Introduction

Depression is one primary public healthcare issue, as one of the leading causes of mental disability, it has gradually become a major contributor to psychological diseases globally, especially during the COVID-19 pandemics. Currently, the population with depression in 2019 was estimated to be 0.35 billion, unfortunately, less than 10% of people with depression treated [1]. It has the tendency to become the world's second-largest disease by 2021, and so far, suicide victims are as high as one million every year. In China, depressed patients have been as high as 90 million [2]. In particular, detecting depression in the early stages, so that how to ensure the early stage correct detection of the patients to get hospital treatment becomes a main research direction in the field. Up to the end of 2019, the monthly active users of Weibo reached 516 million, a net increase of approximately 54 million compared to 2018, of which mobile accounts for 94% of total and daily active users reached 222 million [3]. A large number of Weibo users and rich data provide favorable value for the recognition of certain groups. As a social networking tool in China, Weibo is one of the most popular personal and media publishing platforms. Therefore, this study uses data on Weibo to analyzes the feasibility of detecting depression.

The most widely used methods of depression detection are based on the standard manuals ICD-10 and DSM-5 and the doctor’s clinical experience. The standard manuals are lacking precision or that there is room for incorrect personal diagnosis. The various machine learning techniques are employed for depression detection, there are still some challenges.

- Data are labeled by a single social network is limited, and the training data has a significant influence on the training effect of the machine learning model, so the amount of data needs to be expanded while ensuring data security.
- The increasing number of depressed people puts forward higher requirements on the accuracy of the model.
- With the development of big data, privacy-preserving has attracted attention from the public. How to conduct model training on massive data is an important issue to be solved under the conditions of improving data security and ensure the efficiency of the model.

Federated learning is a newly boomed machine learning framework that can effectively help multiple institutions build a global model under user privacy preservation requirements [4]. In the context of above-mentioned challenges, we aim to develop a federated learning scheme to detect depression. The main contributions in this paper are as follows:

- We propose a new asynchronous federated optimization algorithm with provable convergence for non-convex problems under Weibo users’ data.
- We show that our proposed method can effectively protect users' privacy under the premise of ensuring the accuracy of prediction.
- We use 900 users to train the model together, which improves the utilization of resources and the performance of the model.
- The proposed algorithm can reduce communication overhead.

The rest of this paper is organized as follows. Related literature for current work is presented in “Related work”. In “Methods”, the details of the proposed method are described. “Experiments” evaluates the performance of CAFed in terms of accuracy and convergence for different frameworks, followed by a discussion in “Discussion”. “Conclusion and future work”, concludes the paper and points out potential future research directions.

## Related work

### Machine learning

Using social networks for depression detection has become one of the influential approaches [5], however, designing a useful feature sometimes is not straightforward, especially in the presence of a large amount of data. De Choudhury et al. [6] have measured behavioral attributes relating to social engagement, emotions, linguistic styles and network structure. They leverage these behavioral attributes to build a SVM classifier that provides estimates of the risk of depression. However, this method employs crowdsourcing to compile a set of Twitter users, the effort and time spent are not taken into account. Islam et al. [7] propose three types of properties (emotional, temporal, linguistic style), they then apply machine learning approaches such as Decision Tree, KNN, SVM and Ensemble classifier to study each type independently. Their results show that the Decision tree gives a better outcome. Moreover, they focus on analyzing the time patterns of Facebook users. Unfortunately, this study does not identify who the sufferers are and only detects the Facebook comments for depression detection.

Mcmahan et al. [8] have applied natural language processing (NLP) techniques to develop a depression algorithm for the Thai language on Facebook. They extract from the Facebook user behavior features, including a number of posts, interaction with others, privacy settings, and daytime posting. Considering the limited personal information that can be collected, the behavioral attributes obtained by the algorithm do not cover all relevant factors.

Al-Mosaiwi et al. [9] have proposed a novel model based on user-generated content. They use SVM and Naive Bayes to create a model. The most important is that this model can classify the patients into one out of four levels (Minimal, Mild, Moderate, or Severe depression). Different users may have different behaviour on two social networks, and different regions have different linguistic style, it leads to the accuracy is lower than other methods.

Most of the recognition models either act as black boxes or unsupported incremental learning. It is difficult for AI to classify incremental sequence data. Burdisso et al. [10] have shown a novel white-box text classifier to detect depression. They not only build a public dataset but design a new evaluation metric.

Deep learning has made great significant achievements in image and speech recognition. In traditional text classification, if the text is transformed into a word vector, it will often cause the curse of dimensionality. There are some methods to compress dimensions, these methods do not consider context information in the training process of word vectors and are not satisfactory. Therefore, some researchers apply deep learning to solve large-scale text representation, and then use network structures such as CNN or RNN to automatically obtain feature representation [10–12].

Some researchers [11] have studied the convolution kernels of different sizes to extract multiple features in CNN, which generates the penultimate layer and pass into the fully connected layer. Finally, the probability of each class is predicted by the softmax function. The researchers firstly apply CNN for text classification, the proposed system is composed of four parts: input layer, convolution layer, pooling layer and full connection layer. CNN has obtained better results, although the performance may be slightly worse than RNN on some data sets, the training efficiency of CNN is higher. The model performance is very sensitive to the parameter in neural networks, the parameters adjustment of the neural network is further illustrated in [10].

Wu et al. [13] used recurrent neural networks to compute the posts representation of each user's posts representation. They combine the word representations with other content-based, behaviour and living environment features to build deep neural networks. Trotzek et al. [14] have utilized different word embeddings for training the convolutional neural network and comparing it to a classification based on user-level linguistic metadata. Their experimental results show that an ensemble of both approaches achieves a good performance. Ding et al. [12] presented text-level mining of Sina Weibo data from college students, they use a deep neural network to extract features and propose a deep integrated support vector machine (DISVM) algorithm to detect depression. This approach makes the recognition model more stable and improves accuracy.

### Natural language processing

More and more people like social networks such as Weibo, and post, photos and comments on various information on Weibo. It is possible to analyze and detect whether the users have depression tendency from the text information [10]. To translate the information into input vectors available to the classifier, there are two methods to represent words such as one-hot vector representation and distributed representation. In distributed representation, the words are represented in low dimension. Unlike one-hot vector representation, similar features can have similar vectors in distributed representation, and its computational speed is faster than one-hot vector representation. We use a distributed representation of the words in this work. There are many ways to obtain the distributed representation of the words, we use the pre-trained distributional word embeddings of word2vec [15]. There are two methods to train word embeddings in word2vec: continuous bag of words (CBOWs) and skip-gram. The context in CBOWs predicts target words. In contrast, context is predicted by the target words in skip-gram. We use Weibo corpus Weibo word embeddings which are trained with 300 dimensions [16].

### Federated learning

**Fig. 1:**
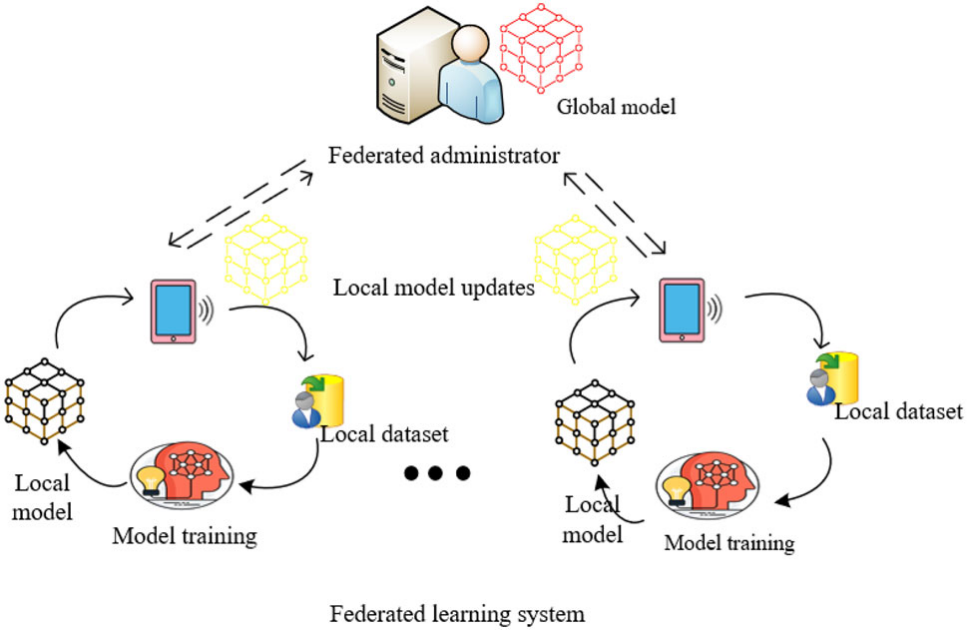
Architecture for Federated learning system: ①: sending encrypted gradients/model to federated administrator. ②: secure aggregation. ③: sending global model to workers. ④: local model updates

Online social networks provide more convenient communication among people, but it also exposes sensitive information about users, the third parties may leak such information. In this paper, we have considered data security and privacy-preserving. Federated learning is a distributed machine learning framework that can effectively solve the problem of data islands, and allow participants to achieve AI collaboration based on not sharing data [4]. Figure 1 shows the federated learning framework, it is composed of server and workers, whose system is similar to parameter servers, but on-device federated learning training tasks are allowed to be executed only when the device is idle, charging, and connected to unmetered networks [17]. Thus, compared to typical distributed machine learning, communication in federated learning needs less costs. Federated learning should handle training data with the following characteristics:

- Non-IID: there are different data distributions on each device [18], i.e., the overall distribution cannot be learned from data on a single device.
- Imbalanced data: Data can be biased to certain labels [17], e.g., users may have different habits or edge devices are monitoring different locations.
- Heterogeneity: Data size and device performance may vary on different local devices [19].

Previous federated learning algorithms such as federated averaging [8] can only train hundreds of devices, but there are many devices in our daily lives. Too many devices training at the same time would congest the network. What’s more, different devices have different computational capacity and battery time, and it is difficult for selected devices to synchronize training [18]. Finally, not all devices participate in training tasks, it only selects the subset of available devices. If the number of survived devices is too small, the server must drop the entire epoch.

Xie et al*.* [20] proposed a novel asynchronous federated optimization algorithm. They apply a mixing hyperparameter to control the tradeoff between the convergence rate and variance reduction and update the global model by weight averaging. However, it is hard to find a suitable mixing hyperparameter. In addition, FedAvg cannot handle incrementally increased data on devices and data/system heterogeneity that leads to stragglers or dropouts. Therefore,Chen et al. [21] proposed an asynchronous online federated learning framework, in which the local model performances online learning with the continuous streaming local data. What’s more,the framework updates the global model in an asynchronous manner to solve the challenges related to different computational loads at heterogeneous edge devices.

There are no well-known studies that have combined federated learning with depression analysis. To overcome the above weakness, we aim to detect depression from Weibo posts. A novel asynchronous federated learning algorithm is proposed to prevent user privacy leaked.

In this paper, we describe in detail the asynchronous federated optimization algorithm based on CNN [22]. Compared with machine learning, the distributed detection model under the federated learning mechanism has dramatically improved the convergence speed and data security, while also ensuring the accuracy of classification.

There are usually words in the collected posts that are not meaningful to our research, such as “this”, “that”, “there”, “oh”. So, we processed the collected posts by using the Harbin Institute of Technology's deactivation vocabulary [23] to remove these meaningless words.

The effect of the word vector is related to the size of the corpus, and the corpus of processing task is not enough to support our experiment, we applied a large—scale microblog word vector trained by word2vec model that was published on the Internet [16, 24]. To ensure the reliability of the training results, the features must be normalized to the range of [0, 1]. The advantage of data normalization is that it can eliminate the differences among different dimensions and make data of different dimensions comparable after normalization (Table 1).

**Table 1:** Notations and terminologies

Notation/tern	Description
$\textbf{n}$	Number of devices
$T$	Number of global epochs
${H}_{\tau }^{i}$	Number of local iterations in the ${\tau }^{th}$ epoch on the $i$th devices
${w}_{t}$	Global model in the ${t}^{th}$ epoch on sever
${w}_{t}^{k}$	The ${k}^{th}$ entry of ${w}_{t}$
${w}_{\tau ,h}^{i}$	Model initialized from ${w}_{\tau }$, updated in the $h$th local iteration, on the $i$th device
${w}_{back}^{i}$	the model before ${w}_{\mathrm{new}}^{i}$ is updated, on the $i$th devices
$c$	A hyper-parameter
$r$	A random number
$\vartheta$	A small constant
$\beta$	A magnitude coefficient
${D}^{i}$	Dataset on the $i$th device
${z}_{t,h}^{i}$	Data(minibatch) sampled from ${D}^{i}$
$\gamma$	Learning rate
Server	The place where the training data are placed
Worker	One worker on each device, process that trains the model

### Differential privacy

We develop the asynchronous federated learning to protect user privacy leakage, the parameters generated in the algorithm will expose intermediate plaintext contents. Once the model parameter is leaked, some important information may be breached [25, 26].

To solve the problem, researchers proposed the concept of differential privacy (DP) [27]. Differential privacy constitutes a strong standard for privacy protection of algorithms on aggregated databases, it does not require special attack assumptions, nor does it pay attention to the background knowledge of the attacker, so it can ensure that the model does not expose whether a data point is used during training. In the federated averaging scheme, the local models are averaged by the server after each epoch of communications, some researchers apply the randomized mechanism to change the global values [28]. The contribution made by each client is hidden in the model aggregation.

#### Definition 1

(*A random mechanism* $$M)$$:$$D\to R$$, with domain and range $$R$$ satisfier $$(\epsilon ,\delta )$$- differential privacy, if for any two adjacent inputs $$d,{d}^{^{\prime}} \in D$$ and for any subset of outputs $$S\subseteq R$$ it holds that $$P\left[M\left(d\right)\in S\right]\le {e}^{\epsilon }\mathrm{Pr}\left[M\left({d}^{^{\prime}}\right)\in S\right]+ \delta $$. In this definition, $$\delta $$ accounts for the probability that plain $$\epsilon -\mathrm{differential}$$ privacy is broken [28].

## Methods

### Initial model

The initial model is one of the important components in the framework of the CAFed depression detection system. Our model is inspired from [29]. The layers present in our CNN architecture are: embedding layer, convolution layer, pooling layer, dropout layer, fully connected layer, and output layer.

**Fig. 2:**
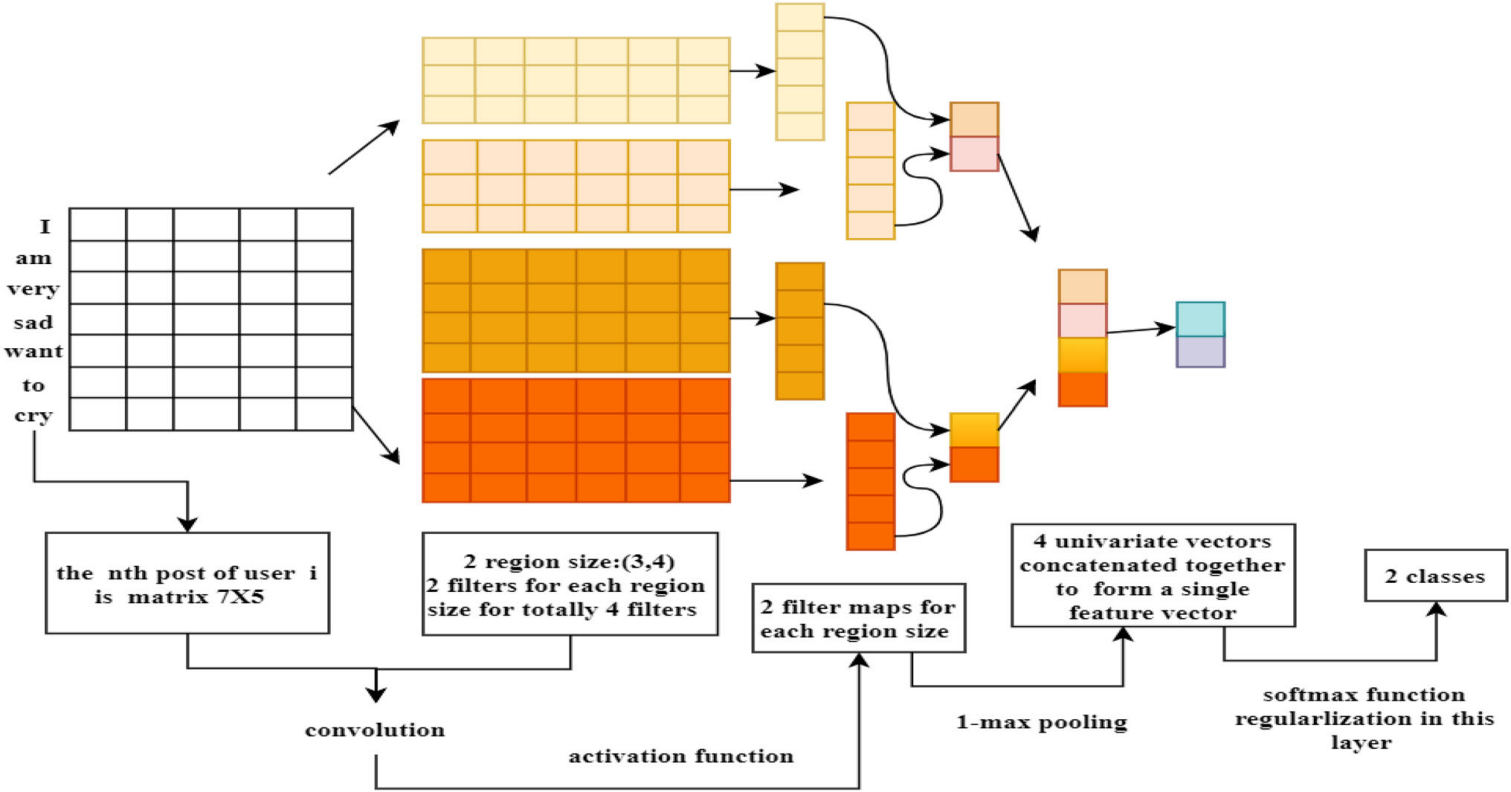
Structure of the text-CNN

The Weibo vector is formed by concatenating all posts of the user. If the dimension of the word vector is $d$ and the length of the $n$th post of a user ${u}_{i}$ is ${l}_{i,n}$ then the dimension of the word vector matrix is ${(l}_{i,1}\cup {l}_{i,2}\cdots {l}_{i,n})\times d$. This word vector matrix is input to the first layer of CNN as shown in Fig. 2. Supposing the dimension of the word vector is ${\varvec{d}}$ and the length of the ${\varvec{n}}$th post of user ${{\varvec{u}}}_{{\varvec{i}}}$ is ${{\varvec{l}}}_{{\varvec{i}},{\varvec{n}}}$ then the dimension of word vector matrix is ${({\varvec{l}}}_{{\varvec{i}},1}\cup {{\varvec{l}}}_{{\varvec{i}},2}\dots {{\varvec{l}}}_{{\varvec{i}},{\varvec{n}}})\times {\varvec{d}}$.

${\mathrm{term}}_{{l}_{i,n}}^{j}$ is denoted as the $j$th word in the $n$th post of a user ${u}_{i}$. We present the $n$th post using ${l}_{i,n}$ words:

<${\mathrm{term}}_{{l}_{i,n}}^{1},{\mathrm{ term}}_{{l}_{i,n}}^{2},\dots {\mathrm{term}}_{{l}_{i,n}}^{{l}_{i,n}}$>. $l$ is comprised of the sequence of words: $<{\mathrm{term}}_{{l}_{i,1}}, {\mathrm{term}}_{{l}_{i,2}},\dots {\mathrm{term}}_{{l}_{i,n}}>$. Then, the word vector is represented as

1 $${W}_{v }{=w}_{ {l}_{i,1}} \circ {w}_{{l}_{i,2}} \circ {w}_{{l}_{i,3}}\dots \circ {w}_{{l}_{i,n}}$$

where ${\mathrm{term}}_{{l}_{i,n}}$ is the words of $n$th post of the user ${u}_{i}$,${w}_{{l}_{i,n}}$ represents the word embedding vector of ${\mathrm{term}}_{{l}_{i,n}}$, and the symbol “$\circ$” is the concatenation operator. Next layer is the convolution layer. In this work, rectified linear units (ReLU) are used to get the convolution feature maps, and the filter length can be 3, 4, or 5. We apply maximum pooling to extract the most important feature and then concatenate values in all feature maps to obtain the pooling layer's final feature vector. To avoid overfitting, we add a dropout layer. Next layer is a fully connected layer. Finally, the sigmoid activation function is applied to classify the given Weibo users.

We fine-tuned the model by the following methods [23], respectively:

- CNN-rand: all words are randomly initialized and then modified during training.
- CNN-static: all words presented by pre-trained word vectors, including the unknown ones that are randomly initialized are kept static, and only other parameters of the model are learned.
- CNN-nostatic: similar to above but the pre-trained vectors are fine-tuned.

### FedAvg model

The federated averaging framework comprises two main parts: server optimization and local training.

First, the server sends the latest model to the client, and the client starts updating the model with local data and uploads the parameters updated to the server. The server aggregates the received parameters, when a certain number of clients are satisfied, generates the latest model, and repeats the steps until the model convergence or the maximum number of iterations is reached. The detailed FedAvg is shown in Algorithm 1.

**Figure:**
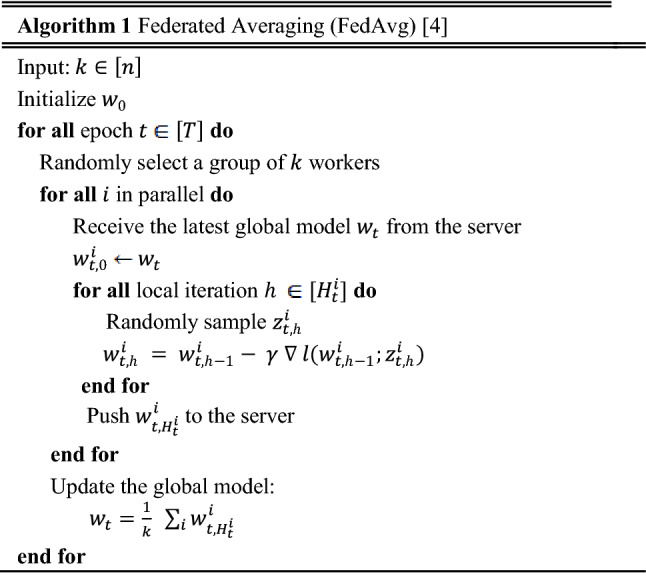

### CAFed model

The asynchronous federated learning framework comprises two main parts: local training and global training.

**Fig. 3:**
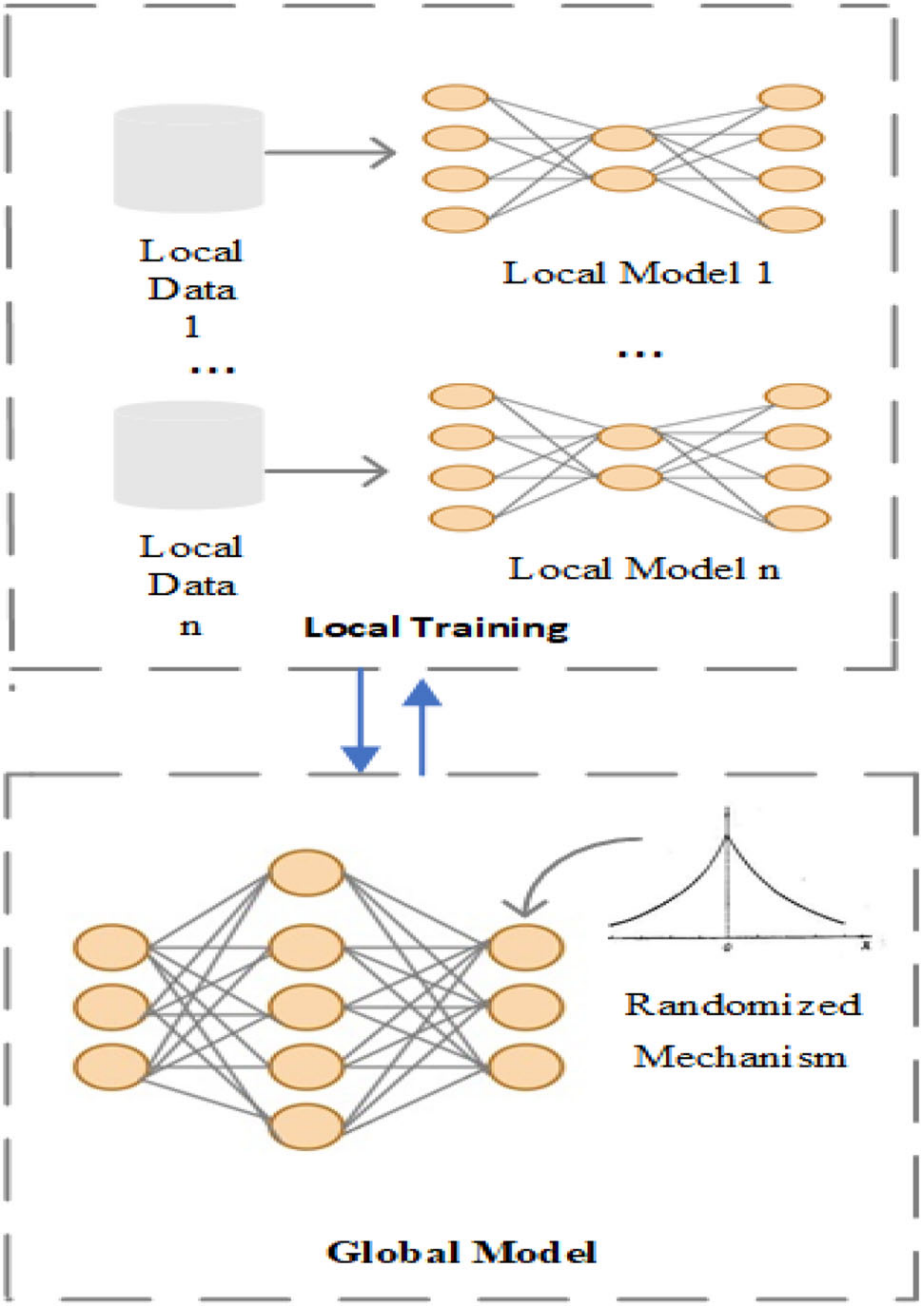
Architecture for CAFed system: the devices use the local data to train the model, after receiving the parameters transmitted by the server. Differential privacy is added before global model updated on the server

During the local training, each device updates the local model according to the global model sent by the server. The server updates the global model immediately after receiving the parameters uploaded by any device. Different from the federated average algorithm, noise is added before updating the global model to protect the parameters from leakage. Figure 3 shows a flowchart of CAFed model.

We consider federated learning with clients (devices) in a horizontal federated learning system, ${D}^{i}$ is the local data on the $i$ th device. ${Z}^{i}$ is a sample from the $i$ th device. The overall objective is to train a global model using the distributed local models from all the devices. To do so, we consider Eq. (2) as the final goal of our optimization.

2 $$\mathrm{min}F(w)=\mathrm{min}\left(\frac{1}{n} \sum\limits_{i\in \left[n\right]}{E}_{{z}^{i}\sim {D}^{i}}\left(w;{z}^{i}\right)\right)$$

A simple premise of federated optimization has global epochs, the server receives a local model ${w}_{new}^{i}$ from worker $i$.

3 $${g}_{t}={w}_{back}^{i}-{w}_{new}^{i}$$

4 $${w}_{t+1}^{k}= {w}_{t}^{k}-{ \partial }_{k} {g}_{t}^{k}$$

The detailed algorithm shows in Algorithm 2. Given that most devices are edge devices, gradients cannot be directly uploaded to the server. We upload the device’s updated model to the server and extract the ${w}_{\mathrm{back}}^{i}$ from ${w}_{\mathrm{back}}$. We save every model that $i$th device uploads to the server in ${w}_{\mathrm{back}}$, ${w}_{\mathrm{back}}^{i}$ is the model before ${w}_{\mathrm{new}}^{i}$ is updated, on the $i$th device, so that server can calculate the gradient that $i$th device updated.

**Figure:**
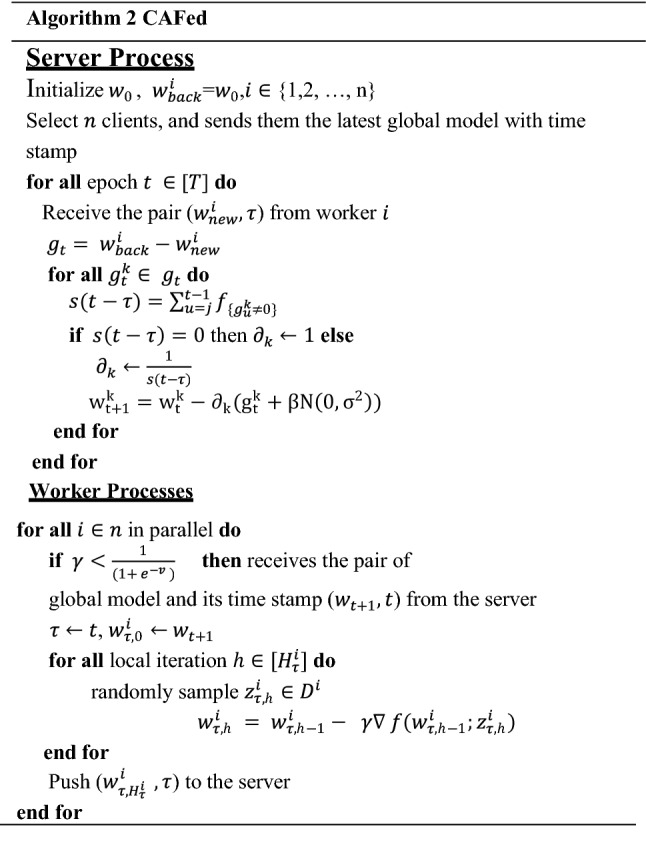

Due to the unreliable connection and low communication efficiency of edge devices, we use Eq. (3) to indirectly calculate the gradient of the device $i$, so as to avoid the adverse impact of the direct upload gradient on the server. Equation (4) represents the global model updated after the server receives the local model.

#### Remark 1

Intuitively, large staleness results in a greater error when updating the global model. When one device pulls $w$, it receives the associated timestamp $\tau$, and updates the $w.$ Meanwhile, intermediate updates (pushed by other devices) increase the timestamp of the server to $t$. Our solution is spired from [25]. Instead of computing the same staleness for each ${g}_{t}^{k}$ of the update ${g}_{t}$, we define an adaptive staleness for each parameter $w$ as Eq. (5):

5 $$s\left(t-\tau \right)= \sum\limits_{u=\tau }^{t-1}{f}_{\left\{{g}_{u}^{k}\ne 0\right\}}$$

where ${f}_{A}$ is the indicator function of condition *A* equal to 1 if condition A is true and 0 otherwise. As shown in Eq. (6), The staleness $s(t-\tau )$ is computed individually for each parameter by counting the number updates applied to it since the last pull by the worker.

6 $${\partial }_{k}^{i}=\left\{\begin{array}{c}\frac{1}{s\left(t-\tau \right)}, s\left(t-\tau \right) \ne 0\\ 1, {\text{otherwise}}\end{array}\right.$$

**Fig. 4:**
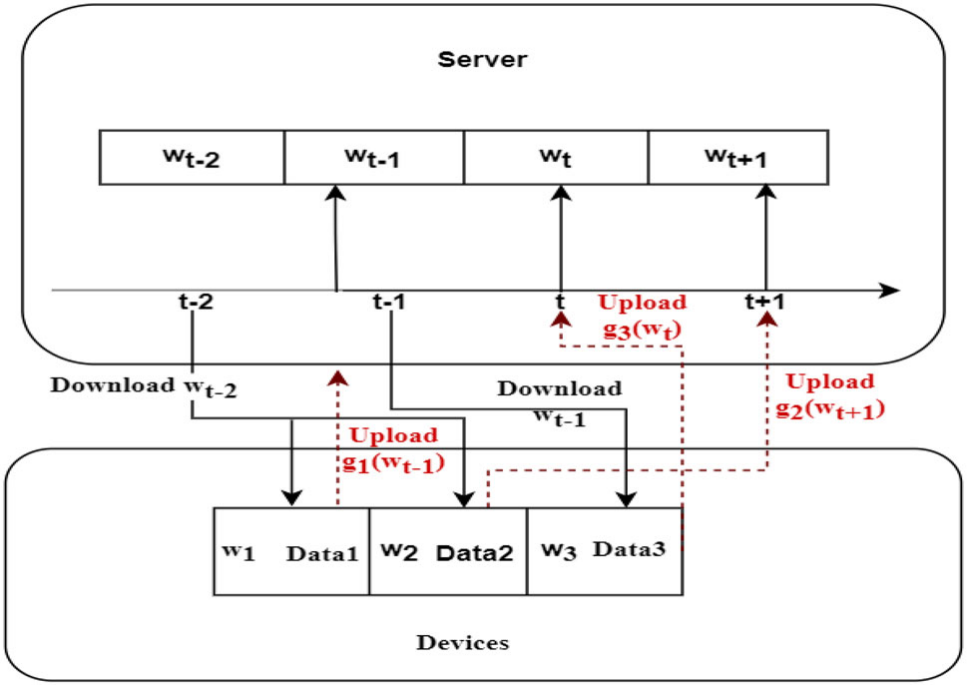
Updating Process of Global Model. At time *t* − 2, the server distributes model ${w}_{t-2}$ to the device 1 and device 2. Then, these two devices start to train their models. At time *t* − 1, device 1 finishes its local training and uploads its local model ${g}_{1}\left({w}_{t-1}\right)$ to the central server for aggregation

The communication is critical in federated learning, to reduce bandwidth consumption, we attempt to deal with the problem by reducing the total number of communications rounds as follows. All devices participating in the training, and the server immediately updates the global model whenever it receives a local model. However, as is shown in Fig. 4 the server can only accept the model from one device for one parameter updated at a time, which means that two or more workers push parameters to the server, the server can only select one of them to update the global model. To improve communication, we choose whether to push at any given time a probabilities choice. When one device wants to push, it generates a pseudo-random number and compares it with other quantities. If the number is larger than that quantity, the data is dropped, otherwise, it is transmitted [25]:

More formally, if one device

7 $$r<\frac{1}{1+{e}^{-v}}$$

where $r\in [\mathrm{0,1}]$ is a random number, $v$ is a hyper-parameter. In practice, the right-hand side of inequation (7) is a Sigmoid function. Thus, the right-hand slide lies in (0, 1).

We apply the randomized mechanism into federated learning [30] a Gaussian white noise with the mean of 0 and the variance of 1 is added to the server process in Eq. (8).

Considering the effect of a randomized mechanism for federated learning, we set a tunable parameter β. The updated model of the server is shown in Eq. (8) (Fig. 4).

8 $${w}_{t+1}^{k}= {w}_{t}^{k}- {\partial }_{k}({g}_{t}^{k}+\beta N (0,{\sigma }^{2}))$$

### Assumptions and lemmas

First, we introduce some definitions and assumptions [31] for our convergence analysis.

#### Definition 2

(*Smoothness*) A differentiable function $f$ is *L*-smooth if for $\forall x,$

9 $$f\left({x}_{k+1}\right)\le f\left({x}_{k}\right)-\frac{1}{2L}{\Vert \nabla \mathrm{f}\left({x}_{k}\right)\Vert }^{2}$$

#### Assumption 1

(*Bounded age*) All staleness variables $s(t-\tau )$ are bounde:

10 $$s(t-\tau )\le M$$

#### Assumption 2

(*Strong convexity*) A differentiable function $f$ is $\mu -\mathrm{strongly}$ convex if for $\forall x$,

11 $$f\left({x}_{k+1}\right)\le f\left({x}_{k}\right)-\frac{\mu }{2}{\Vert \nabla \mathrm{f}\left({x}_{k}\right)\Vert }^{2}$$

#### Assumption 3

Let ${z}_{t}^{i}$ be a sample from the $i$th device’s local data uniformly at random. The variance of stochastic gradients in each device is bounded:

12 $$E {\Vert \nabla {F}_{i }\left({w}_{t}^{i},{z}_{t}^{i}\right)-\nabla {F}_{i }({w}_{t}^{i})\Vert }^{2}\le {\sigma }^{2}\,\mathrm{ for\, k}\hspace{0.17em}=\hspace{0.17em}1, \ldots , n.$$

#### Lemma 1

(*Results of one step SGD*) Assume Assumption 1 and 2, that

${p}_{i}$ is the weight of the $i$th device.

If $\gamma \le \frac{1}{4L}$, we have

13 $$\begin{aligned} {\varvec{E}}{\Vert {v}_{t+1}^{-}-{w}^{*}\Vert }^{2} & \le \left(1-{\eta }_{t }\mu \right) E{\Vert {w}_{t}^{-}-{w}^{*}\Vert }^{2} \\ &\quad +{r}^{2}E{\Vert \left({g}_{t}-{g}_{t}^{-}\right)\Vert }^{2}+6L{\eta }_{t}^{2} {\varnothing }\\ &\quad +2E \sum_{k=1}^{n}{p}_{k}{\Vert {w}_{t}^{-}-{w}_{k}^{i}\Vert }^{2}\end{aligned}$$

where $$\varnothing ={F}^{*}-\sum_{k=1}^{n}{p}_{k} {F}_{k}^{*}\ge 0$$

#### Lemma 2

(*Bounding the variance*) Assume the above Assumption 3 holds, it follows that

14 $$E{\Vert \left({g}_{t}-{g}_{t}^{-}\right)\Vert }^{2}\le \sum\limits_{k=1}^{n}{p}_{k}^{2} {\sigma }_{k}^{2}$$

#### Lemma 3

(*Bounding the divergence of* $\left\{{w}_{t}\right\}$), Assume ${\Vert \nabla \mathrm{F}\left({w}_{t}\right)\Vert }^{2}\le {\varepsilon }^{2}$, that ${\eta }_{t}$ is non-increasing and ${\eta }_{t}\le 2{\eta }_{t+E}$ for all $t\ge 0$. It follows that

15 $$E\left[\sum_{k=1}^{n}{\Vert {w}_{t}^{-}- {w}_{k}^{t}\Vert }^{2}\right]\le 4{\eta }_{t}^{2}{(E-1)}^{2}{\varepsilon }^{2}$$

### Convergence guarantees

Based on the above assumptions, we show the convergence rate and how the staleness affects the convergence. With some modification, we adapted further the proof of the convergence by [31–33] and lead to the following theorem, which indicates the convergence rate and our algorithm's linear property of speed-up.

16 $${g}_{t}= \frac{1}{n}\sum_{i=1}^{n}{p}_{i} \nabla l({w}_{t}^{i},{z}_{t}^{i})$$

17 $${g}_{t}^{-}=\sum_{i=1}^{n}{p}_{i}\nabla {F}_{i}({w}_{t})$$

By substituting Lemmas 1 and 2 into Lemma 3, we can get

18 $$E{\Vert {w}_{t+1}^{-}-{w}^{*}\Vert }^{2}\le \left(1-{\eta }_{t }\mu \right)E{\Vert {w}_{t}^{-}-{w}^{*}\Vert }^{2}+B$$

where $$B=\sum_{k=1}^{n}{p}_{k}^{2}{\sigma }_{i}^{2}+6L\phi +8{(E-1)}^{2}{\varepsilon }^{2}$$

Let ${\Delta }_{t }= E{\Vert {w}_{t+1}^{-}-{w}^{*}\Vert }^{2}$, from lemma 1, lemma 2 and lemma3, it follows that

$${\Delta }_{t+1}\le \left(1-{\eta }_{t}\mu \right){\Delta }_{t}+{\eta }_{t}^{2}B$$

For a diminishing step size, ${\eta }_{t}=\frac{\beta }{t+\gamma }$, it is discretionary, so $\beta$ and $\gamma$ need to ensure ${\eta }_{1}=\frac{\beta }{1+\gamma }\le \frac{1}{4L}$ and$\frac{\beta }{t+\gamma }\le 2$. We prove${\Delta }_{t}\le \frac{\upsilon }{\gamma +t}$, where $\upsilon =\mathrm{max}\left\{\frac{{\beta }^{2 }B}{\beta \mu -1}, \left(\gamma +1\right){\Delta }_{1}\right\}$

$${\Delta }_{t+1}\le \left(1-{\eta }_{t}\mu \right){\Delta }_{t}+{\eta }_{t}^{2}B$$

$$=\left(1-\frac{\beta \mu }{\gamma +t}\right)\left(\frac{\upsilon }{t+\gamma }\right)+ \left(\frac{{\beta }^{2}B}{{(t+\gamma )}^{2}}\right)$$

$$=\frac{t+\gamma -1}{{(t+\gamma )}^{2}}\upsilon +\left[\left(\frac{{\beta }^{2}B}{{\left(t+\gamma \right)}^{2}}\right)-\left(\frac{\beta \mu -1}{{\left(t+\gamma \right)}^{2}}\right)\upsilon \right]$$

$$\le \frac{\upsilon }{t+\gamma +1}$$

Then by the strong convexity

$$E\left[F\left({w}_{t}^{-}\right)\right]-{F}^{*}\le \frac{L}{2}\frac{\upsilon }{t+\gamma }$$

Completing the proof.

From the perspective of the sever, the update procedure of parameter $\mathrm{w}$ for Algorithm 2 can be written as1920
where ${w}_{\tau ,h,k}^{i}$ denotes $k$th parameter initialized from ${w}_{\tau }$, updated in the $h$th local iteration, on the $i$th device. We use the staleness sequence{${s}_{t}$}, *t* = 0, 1, …, *T* in Algorithm 2. Where ${s}_{t}$ is the sum of staleness for all parameters on the $i$th device.

Note that $\gamma$ is modulated in (19), one can verify that the convergence rate is faster than $\mathrm{in}$ (16). From Theorem 1 proposed by Lei et al. [34] with some modification, we have the following theorem, which indicates the convergence rate and our algorithm's linear speedup property.

#### Theorem 1

Let *C*_1_, *C*_2_, *C*_3_, *C*_4_ be certain positive constants depending on the loss function $L(\mathrm{w})$. Under certain commonly used assumptions proposed by Lei et al. [34] and the theorem proposed by [35], we can achieve a convergence rate of

21 $$ \frac{1}{{\sum\nolimits_{{t = 1}}^{T} {1/s_{t} } }}\sum\limits_{{t = 1}}^{T} {\frac{1}{{s_{t} }}} E\left( {\left\| {\nabla L\left( {w_{t} } \right)} \right\|^{2} } \right) \le 2\frac{{\sqrt {\frac{{2c_{1} c_{2} }}{{z_{{\tau ,h}}^{i} }}\sum _{{t = 1}}^{T} \left( {\frac{1}{{s_{t} ^{2} }}} \right)} }}{{\sum\nolimits_{{t = 1}}^{T} {\frac{1}{{s_{t} }}} }} $$

where *T* is the total epoch number, if

22 $$\upgamma =\sqrt{\frac{{c}_{1 }{z}_{\tau ,h}^{i} }{\sum_{t=1}^{T}\left(\frac{2}{{{\mathrm{s}}_{\mathrm{t}}}^{2}}{\mathrm{c}}_{2}\right)}}$$

under the prerequisite that

23 $$\gamma \le \frac{ {c}_{2}}{{c}_{3}{ s}_{t} \sum_{j=t-M}^{t-1} \frac{1}{{\mathrm{s}}_{\mathrm{j}}^{2}}},\forall t\in [M,T]$$

and

24 $${c}_{3}\frac{\gamma }{{s}_{t}} + {c}_{4} M\frac{{\gamma }^{2}}{{ s}_{t}} \sum_{k=1}^{M}\frac{1}{ {s}_{t+k}}\le 1,\forall t$$

When selecting small enough $\gamma$, Eqs. (19) and (20) can always be satisfied, it means that the LHS of (21) is guaranteed to converge. We also note that the relationship between staleness and convergence rate can be found by observing the RHS (21).

#### Remark 2.

We found that the RHS of (21) is of the form $h\left({x}_{1,\cdots ,}{x}_{T}\right)=O\left(\frac{\sqrt{{x}_{1}^{2}+{x}_{2}^{2}\cdots {x}_{T}^{2}}}{{x}_{1}+{x}_{2}+\cdots {x}_{T}}\right)$[35] by letting${x}_{t}=\frac{1}{{s}_{t}}$. If ${x}_{1}={x}_{2}=\cdots ={x}_{T}$, $h$ is minimized. We consider the ideal assumption of the staleness, namely, we take ${s}_{t}$ as a constant$s$, we have

25 $$\frac{1}{T}\sum_{t=1}^{T}E\left({\Vert \nabla L\left(w\right)\Vert }^{2}\right)\le 2 \frac{\sqrt{2{c}_{1 }{c}_{2 } }}{\sqrt{ {z}_{\tau ,h}^{i} T}}$$

Thus, the convergence rate is roughly $O(1/\sqrt{{z}_{\tau ,h}^{i} T})$, where $T$ is the total number of epochs, ${z}_{\tau ,h}^{i}$ denotes the mini-batch size on the $i$th device. We can draw a conclusion that a linear speedup can be achieved in our Algorithm 2, according to Theorem 1 from [30].

Proof. from (24) we have

$${c}_{3}\frac{\gamma }{{ s}_{t}} + {c}_{4} M\frac{{\gamma }^{2}}{{ s}_{t}} \sum_{k=1}^{M}\frac{1}{ {s}_{t+k}}$$

$$={c}_{3 {H}_{\tau }^{i} \frac{\gamma }{{ {H}_{\tau }^{i} s}_{t}}}+{c}_{4}({z}_{\tau ,h}^{i}{)}^{2} M\frac{\gamma }{{z}_{\tau ,h}^{i}{ s}_{t}}\sum_{k=1}^{M}\frac{\gamma }{ {z}_{\tau ,h}^{i}{ s}_{t+k}}$$

$$\le 1$$

With (22) we have.

26 $$\frac{{\gamma }^{3}}{{z}_{\tau ,h}^{i}{ s}_{t}} {c}_{3}\sum_{j=t-M}^{t-1} \frac{1}{{s}_{j}^{2}}\le \frac{{\gamma }^{2}}{{z}_{\tau ,h}^{i} {s}_{t}^{2}} {c}_{2},\forall t$$

Note that the upper bound of staleness is *M* in our setting. Then it follows from Theorem 1 proposed by [30] that

$$\frac{1}{\sum_{t=1}^{T} 1/s_{t}} \sum_{t=1}^{T}\frac{1}{{s}_{t}}E\left({\Vert \nabla L\left({w}_{t}\right)\Vert }^{2}\right)$$

$$\le \frac{{c}_{1}+ \left(\sum_{t=1}^{T} \frac{{\gamma }^{2}}{{z}_{\tau ,h}^{i}{ s}_{t}^{2}} {c}_{2}+\frac{{\gamma }^{3}}{{{z}_{\tau ,h}^{i} s}_{t}} {c}_{3}\sum_{j=t-M}^{t-1} \frac{1}{{s}_{\mathrm{j}}^{2}}\right)}{\sum_{t=1}^{T} \frac{\gamma }{{s}_{t}}}$$

$$\le \frac{{c}_{1}+{\gamma }^{2}\sum_{t=1}^{T}\frac{2}{{{z}_{\tau ,h}^{i} s}_{t}^{2}} {c}_{2}}{\sum_{t=1}^{T}\frac{\gamma }{{s}_{t}}}$$

$$= \frac{ 2\sqrt{{c}_{1}\sum_{t=1 }^{T}\frac{2}{{{z}_{\tau ,h}^{i} s}_{t}^{2}} {c}_{2}}}{\sum_{t=1}^{T}\frac{1}{{s}_{t}}}$$

$$= \frac{2 \sqrt{\frac{2{c}_{1}{c}_{2}}{{z}_{\tau ,h}^{i}} {\sum }_{t=1}^{T}\left(\frac{1}{{s}_{t}^{2}}\right)}}{\sum_{t=1 }^{T}\frac{1}{{s}_{t}}}$$

completing the proof.

## Experiments

### Data analysis

We experimented on Weibo user’s posts for depressive behavioural exploration and detection. Datasets were collected from the social network. The greatest challenge for data collection is to find positive class among Weibo users. Some researchers issue survey/questionnaires to users, but it is not sure that the answers given by patients to the questionnaire are accurate. De Choudhury et al. [32] have employed crowdsourcing to collect comments from several hundred Twitter users who report that they have been diagnosed with clinical MDD using the CES-D2 (Center of Epidemiologic Studies Depression Scale) screening test. However, this method needs to spend money, Crowdsourcing, for example, requires a certain amount of money, so relatively few users are collected.

In this paper, the real users who have experienced medical treatment and non-depression users were screened out through questionnaire consultation. With the permission of the users, we adopted the crawler method to obtain the related Weibo users' data by signing confidentiality agreements and privacy norms. We adopted the crawler method to collect 1000 Weibo users' data, which includes nickname, number of Weibo, number of fans, number of followers, and content of Weibo. We collected a user's data for one year. We also exclude those vague expressions, for example, ‘I seem to have depression,’ or ‘I have been depressed for a long time’ and’ suspected that I was depressed’, etc. We divide the data set into two groups. One group is used to count the posts posted by users, and the other is used to count the number of posts by users, the number of fans, the number of likes, the number of comments, and the number of followers. Details of the experimental data set are shown in Table 2. The evaluation matrices parameters (precision, recall and F-measure) have been used to execute these classifiers. It has been conducted based on four different ways [7]. True Positive (TP) is the depression cases that are positive and anticipated as positive and anticipated as positive. True Negative (TN) is the depression cases that are negative and anticipated as negative. False Negative (FN) is the depression cases that are positive but anticipated to be negative. False Positive (FP) is the depression cases that are negative but anticipated to be positive. All the evaluation metrics are defined as follows.

**Table 2:** Experimental data set introduction

Total sample	Depression samples		Number of normal users	
	Training sample size	Test sample size	Training sample size	Test sample size
900	253	74	467	106

### Experiment results

1. CNN
  In this experiment, the word embedding is trained on the posts, where are 900 users. The word embedding is fine-tuned during training to improve performance. According to our experiments, we set the length of the ordered vector set $L=$ 27,941, the output dimension of word2vec *d* = 300. In addition, we also adjust the batch size which is a parameter used with the Stochastic Gradient Descent (SGD) method in the model training phase. Here, the SGD updates the weights in the CNN after each set of batch size are examined. A large batch size increases the chance of overfitting, while a smaller batch size may result in a longer time for convergence [11]. Therefore, we empirically set batch size = 32, which achieves a good balance among the two factors mentioned above. We use ${l}_{2}$ regulation to avoid overfitting. The other parameters used in our experiments are as follows: the number of filters: 128, dropout: 0.5, and loss function: binary cross-entropy. The results are shown in Table 3.
2. Federated averaging
  We apply the FedAvg to detect depression and use the CNN model of the first experiment as the network structure. Considering the lack of a large enough data set and hardware devices problems, after many experiments, we finally determined that the training set is partitioned onto n = 10 devices, each of the n = 10 partition has 72 Weibo users in each round. For any worker, the minibatch size for SGD is 16. The detailed FedAvg is shown in Algorithm 1 and the results are shown in Table 4.
3. CNN asynchronous federated
  To make a comparison with the previous two experiments, we also adopted the CNN model as the network architecture. The detailed CAFed is shown in Algorithm 2, and the results are shown in Table 5. In each experiment, the training set is also partitioned onto $n =$ 10 devices. We evaluate the magnitude of normal noise in the randomization mechanism on the model. Comparative experiments are conducted to analyze the effect of the magnitude of noise on model performance. The results are shown in Table 6 with both randomized and nonrandomized settings. For the randomized version, four values of magnitude are chosen, i.e., 0.0001, 0.001, 0.01 and 0.05.
4. Experiments on MNIST
  To make our proposed algorithm CAFed more reliable, we evaluate the performance on an extensive training set, composed by the MNIST training dataset ($\mathrm{i}.\mathrm{e}.$, with 60,000 images). The data is shuffled, and then partition into 100 devices, each receiving 600 images. We divided the Non-IID data. Firstly, we sort the data by digital label, divide it into 200 shares of size 300, and assign each of 100 devices 2 shards. This is a pathological Non-IID partition of the data because many devices will only have examples from two digits. Both of these partitions are balanced.

**Table 3:** Evaluation results for CNN

Method	Precision (%)	Recall (%)	F-measure (%)	Accuracy (%)
CNN + rand	91.76	80.88	85.94	86.11
CNN + static	90.01	83.33	86.96	83.33
CNN + nostatic	87.50	100	93.33	87.50

**Table 4:** Comparison results for FedAvg

Method	Precision (%)	Recall (%)	F-score (%)	Accuracy (%)
FedAVg + rand	89.47	85.00	87.18	83.33
FedAvg + static	81.82	81.82	81.82	73.33
FedAvg + nostatic	100	81.82	90.00	86.67

**Table 5:** Comparison results on CAFed

Method	Precision (%)	Recall (%)	F-measure (%)	Accuracy (%)
CAFed + rand	86.90	85.25	73.20	67.92
CAFed + static	80.00	89.75	84.59	80.00
CAFed + nostatic	90.00	81.82	85.26	86.67

**Table 6:** Magnitude of randomization vs. testing accuracy on Weibo

Randomization	CAFed-rand (%)	CAFed-static (%)	CAFed-nostatic (%)
Nonrandomized			
$\beta$ = 0	85.27	80.00	86.67
Randomized			
$\beta$ = 0.0001	83.67	78.00	84.00
$\beta$ = 0.001	81.25	76.27	81.62
$\beta$ = 0.01	78.63	74.00	78.00
$\beta$ = 0.05	73.00	71.23	74.00

Table 7 shows the test set accuracy with different methods. We use a convolutional neural network (CNN) with three convolutional layers and dropout layers followed by one fully connected layer.

**Table 7:** Magnitude of randomization vs testing accuracy on MNIST

Randomization	FedAvg (%)	CAFed (%)
Nonrandomized		
$\beta$ = 0	72.23	83.26
Randomized		
$\beta$ = 0.0001	70.26	81.75
$\beta$ = 0.001	68.14	80.00
$\beta$ = 0.01	67.75	78.13
$\beta$ = 0.05	65.00	76.00

### Analysis of experimental results

**Fig. 5:**
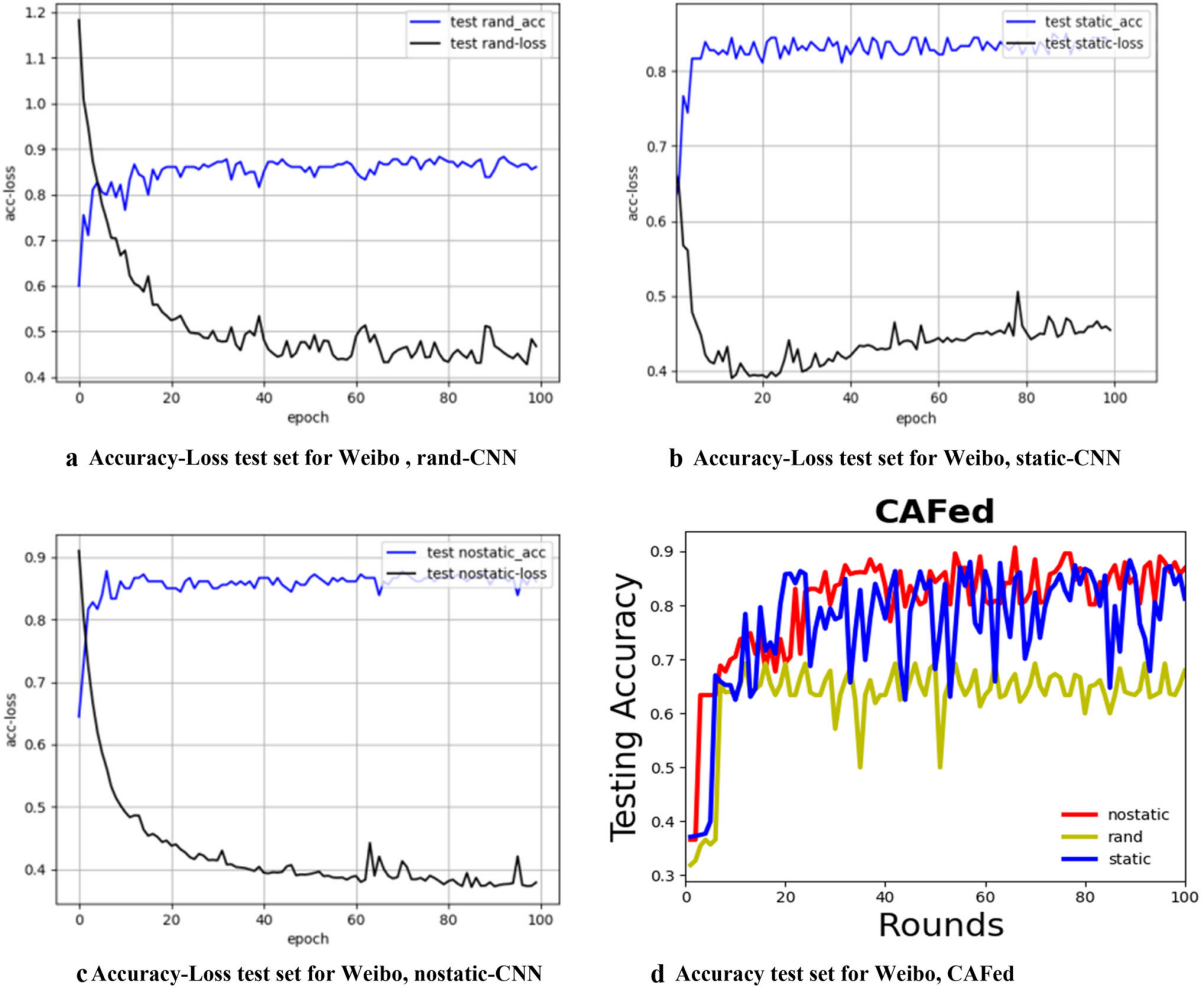
Test set accuracy and convergence for Text-CNN, **a**–**c** use different fine-tuned model. **d** Describes test set accuracy for Algorithm 1, with a different fine-tuned model. Noted that the accuracy of nostatic-CNN is high than other models. Actually, they are centralized frameworks, namely, all participants’ data are sent to a central server, so it is very effective. However, it violates data privacy as all users’ data are exposed to the server

For all the algorithms under investigation, we compute the number of global epochs by counting how many times the global model is updated. The total number of global epochs *T* = 200 in Algorithm 1, and *T* = 100 in Algorithm 2. In Fig. 5, we show how to word embedding type affects the performance of the model. It can be noted that CNN-nostatic is performing better than other models.

**Fig. 6:**
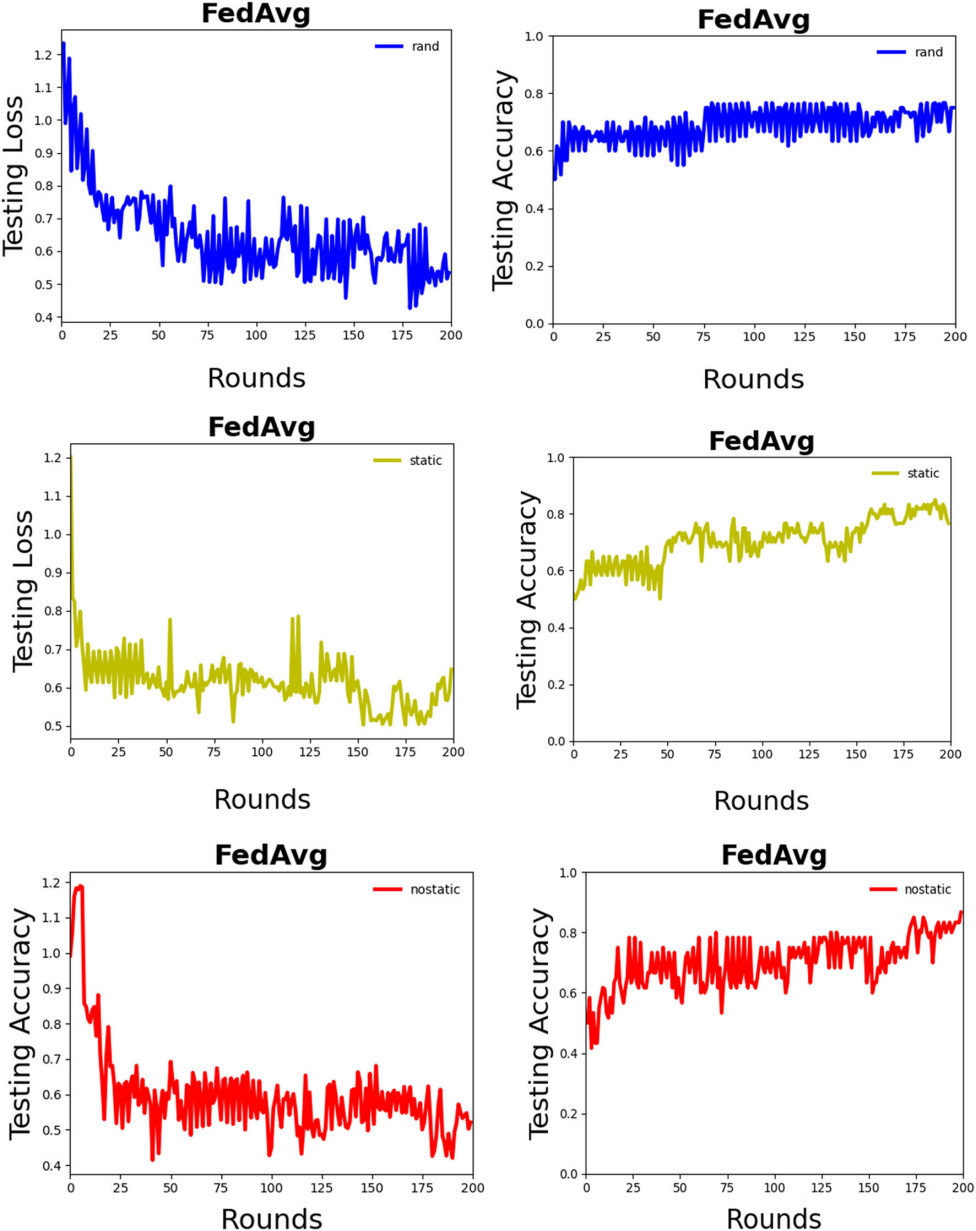
The left column is test set convergence, and the right is the accuracy of test set for Weibo. It is obvious that the performance of FedAvg-nostatic is better than other fine-tuned model

From Fig. 6, we can observe that the performance of the FedAvg-nostatic model is better than other models. At the same time, we find that the curve of the federated average model fluctuates more than that of the machine learning model. We think there are two reasons: first, the data set is not large enough, each device has only a small amount of data, which affects the performance of the model. Second, deep learning method is not concerned with data security and preserve protection. Deep learning method is to pull all data together and use all data to train a model${ M}_{\mathrm{SUM}}$. A federated learning system is a learning process in which the data owners collaboratively train a model ${M}_{\mathrm{FED}}$, in which process any worker does not expose its data to a server or other workers. From the inequality (27) [4], we can conclude that the performance of ${M}_{\mathrm{FED}}$ is not well than ${M}_{\mathrm{SUM}}$. ${\mathrm{ACC}}_{\mathrm{FED}}$ and ${\mathrm{ACC}}_{\mathrm{sum}}$ represent the accuracy of Federated learning and deep learning, respectively. Let $\delta$ be a non-negative real number, if

27 $$\left|{\mathrm{ ACC}}_{\mathrm{FED}}-{\mathrm{ACC}}_{\mathrm{SUM}}\right| < \delta $$

we say federated learning has ${\varvec{\delta}}-{\varvec{a}}{\varvec{c}}{\varvec{c}}{\varvec{u}}{\varvec{r}}{\varvec{a}}{\varvec{c}}{\varvec{y}}$ loss.

**Fig. 7:**
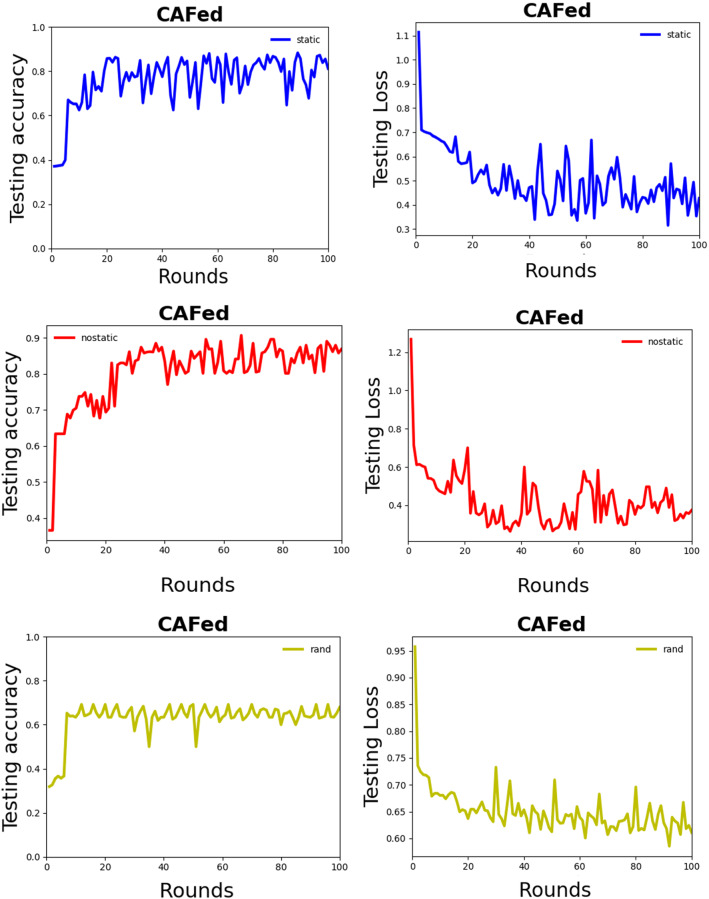
The left column is test set accuracy, and the right is the convergence of test set, with nostatic fine-tuned model. Note that CAFed needs less training rounds and the accuracy of CAFed model is high than FedAvg

From Fig. 7, we can observe that our proposed method is performing better than FedAvg when differential privacy is not added. After the addition of differential privacy, it is obvious that the performance of the model decreases, but the privacy of users is protected. As shown in Table 6, a small noise did not cause a too big effect on the performance of the model. With a large noise, the performance of the model becomes worse. We observed that the difference in accuracy between a no-private and a private model is about 2%, therefore, we should make a trade-off between accuracy and privacy protection.

In Table 7, we observed that the effect of the magnitude on the test accuracy under the different model structure, and because of the added noise, models need more running rounds to converge.

In Figs. 6 and 7, we show CAFed and FedAvg convergence when the number of global epochs grows. Obviously, CAFed converges faster than FedAvg. Because FedAvg has to wait for some devices respond in each epoch, while CAFed only needs one device’s response to move on to the next epoch. What’s more, in each global epoch, FedAvg has more communications compared with CAFed. Overall, with the same amount of communication overhead, CAFed converges faster than FedAvg.

**Fig. 8:**
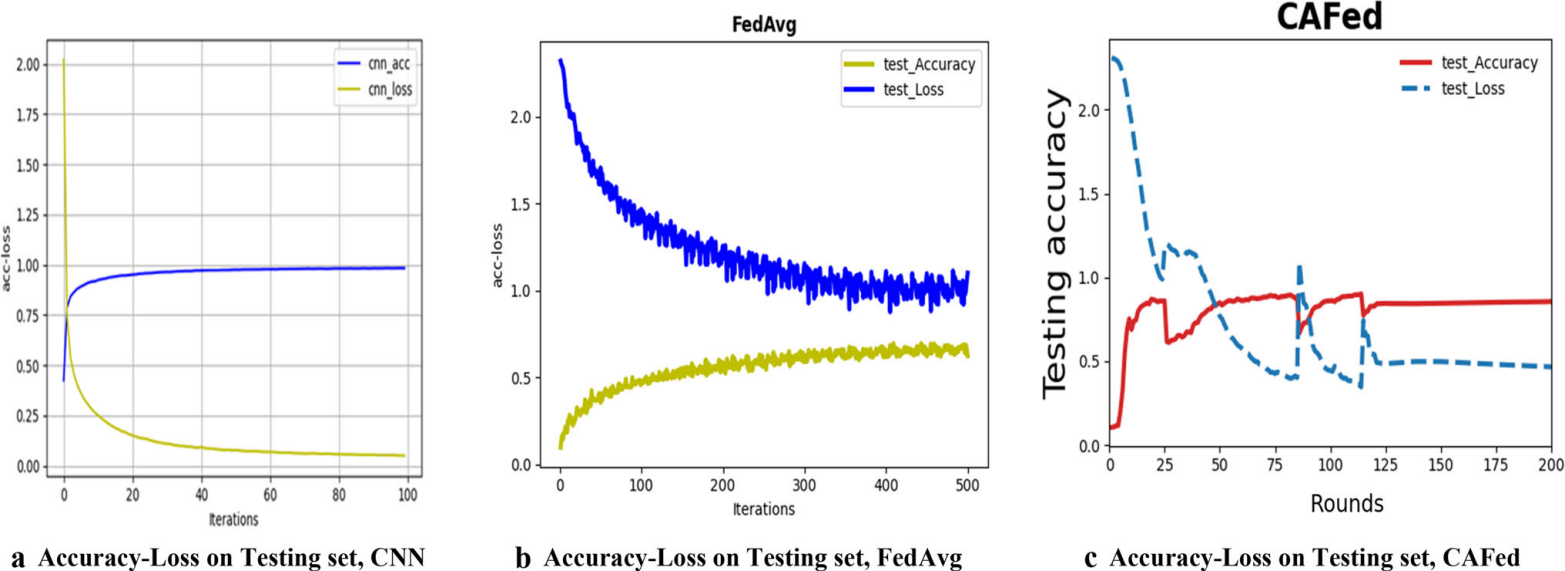
Testing set accuracy-loss for the MNIST, with different fine-united model

In Fig. 8, we show the test set accuracy-loss performance by use of MNIST with different methods. As shown in Fig. 8, CNN makes more progress in each round, however, the performance of the federated average model is not as good as the asynchronous federated model, and more training rounds are needed. Although our model has a higher accuracy than FedAvg, the curve fluctuation is relatively large. It is believed that the global model can be aggregated by the proposed method. The asynchronous federated model updates the global model immediately after receiving any parameter uploaded by the client, however, the performance of the model is affected by the different data quality and device environment of each client. On the other hand, we think that the inclusion of differential privacy has also affected the accuracy of the model.

A comparison among different deep learning frameworks is provided in Table 8. In summary, the centralized framework is very effective with higher accuracy than a distributed framework, but it violates data privacy as all devices’ data are exposed to the server, and if the central server fails, the whole network stops working. In the distributed framework, devices collaboratively train a model by sharing local model updates to avoid privacy leakage.

**Table 8:** Comparative results analysis on Weibo

Frameworks	Technique used	Accuracy (%)	Precision (%)	Recall (%)	F1 score (%)
Centralized framework	CNN + rand	86.11	91.67	80.88	85.94
	CNN + static	83.33	90.01	83.33	86.96
	CNN + nostatic	87.50	87.50	100	93.33
Distributed framework	FedAvg + rand	83.33	89.47	85.00	87.18
	FedAvg + static	73.33	81.82	81.82	81.82
	FedAvg + nostatic	86.67	100	81.82	90.00
	CAFed + rand	67.92	86.90	85.25	73.20
	CAFed + static	80.00	80.00	89.75	84.59
	CAFed + nostatic	86.67	90.00	81.82	85.26

## Discussion

To better understand our proposed algorithm, we apply the following techniques for depression detection, such as text-based CNN technology, FedAvg and CAFed. Table 8 shows the results of various models. It is clearly observed that the nostatic mode performs better than the other modes, it not only uses a pre-trained model, but also continuously adjusts the word vectors during training. The static mode uses a pre-training model, but does not adjust the word vector during the training process; the random mode does not use the pre-training model, but uses random initialization, and then continuously adjust word vectors in the training process. Although the F1 score of the FedAvg algorithm is best in a distributed framework, it usually requires more communication rounds to achieve better results, and CAFed can also achieve the same effect as FedAvg in nostatic mode, and not so many communication rounds. The most important is that CAFed also considers the user's privacy and security, and makes a trade-off between the privacy protection and the accuracy. Systematically, CAFed has the following advantages compared to FedAvg (Federated Average):

1. Convergence rate
  For asynchronous federated learning (Fedasync), when the server receives a local model uploaded by any worker, it updates the global model immediately. Unlike synchronous federated learning (Fedsync), the server needs to wait for a subset of available workers to push parameters before aggregating them. In general, CAFed converges much faster than FedAvg.
2. Communication costs
  All devices participate in training, the server can only accept one device's parameters to update in each global epoch. In order to reduce the communication costs, we only select one of the devices to push, and at the same time, avoid network congestion. Unlike FedAvg, if the number survived devices are too small, the entire global epoch including all the received updates may be dropped by the server.
3. Scalability
  The federated averaging can only process hundreds of devices in parallel at a time, which may also cause network congestion, while the asynchronous algorithm not only does not cause network congestion but can also process many devices. Due to the limited data set we collected; this advantage was not obvious in our experiments.

## Conclusion and future work

In this paper, we propose a novel asynchronous federated optimization algorithm on Non-IID training data. We have proved that a linear speedup can be achieved in our algorithm. As shown in the experiments, the proposed CAFed system outperforms other baselines approaches, we have considered users' privacy. It is regarded to be significant in regard to the recognition of depressed patients without revealing their privacy. After all, no one wants their privacy to be exposed, especially some sensitive information. Moreover, we consider that these results can help develop traditional detection methods, enabling those suffering from depression to be detected and receive treatment as soon as possible.

To better understand the proposed algorithm, we apply text-based CNN technology, FedAvg and CAFed for depression detection in this paper. Table 8 shows the results of various models with different frameworks. It is reasonable that text-based CNN achieves 87.50% of accuracy. Because it applies the traditional data transactions models, specifically, one party collects and transfers data to another party, and other parties will be responsible for cleaning and fusing the data. Finally, a third party will take the integrated data and build models for other parties to use [15]. So, we need to push the data to a third party to ensure the accuracy of the model during the entire data transmission processing. Unlike text-based CNN, the parties did not expose the data to the server or other parties, so the other two models are slightly less accurate compared to the first model. The deep learning technology guarantees the accuracy of the model, it does not consider data security and privacy. Federated learning technology can not only help multiple parties to build a sharing model but also strengthen data privacy and security [13].

We propose a novel asynchronous federated optimization algorithm for Weibo users’ data. It is proved that a linear speedup can be achieved in our algorithm. As shown in the experiments, the proposed CAFed system outperform other baseline approaches, the current system achieved 86.67% recognition rate of depression, which can effectively distinguish between depression and normal individuals in practice. Most importantly, we have considered users' security and privacy. We believe that it is significant to recognise depressed patients without revealing their privacy. These results can help develop traditional detection methods, enabling those suffering from depression to be detected and receive treatment as soon as possible. Our future work includes: (1) Considering behavior-based features as additional information sources to further boost up the performance; (2) taking into account incentive mechanism module to improve the proposed algorithm; (3) extending the proposed approach to effectively detect other important mental illness, such as anxiety and bipolar disorders [36].
